# An Offer You Can’t Refuse: Opportunities for Intraprofessional Collaboration Learning in the Workplace

**DOI:** 10.5334/pme.1863

**Published:** 2026-07-13

**Authors:** Maarten van der Ven, Isa Wijnands, Natasja Looman, Esther de Groot, Cornelia Fluit, Wietske Kuijer-Siebelink, Dieneke van Asselt

**Affiliations:** 1 Geriatric Medicine Department, Radboud University Medical Center, Nijmegen, the Netherlands; 2 Primary & Community Care Department, Radboud University Medical Center, Nijmegen, the Netherlands; 3 Julius Center for Health Sciences and Primary Care, Utrecht University Medical center, Utrecht, the Netherlands; 4 Department of Research on Learning and Education, Radboud University Medical Center Health Academy, Nijmegen, the Netherlands; 5 School of Education, HAN University of Applied Sciences, Nijmegen, the Netherlands

## Abstract

**Introduction:**

Intraprofessional collaboration (IntraPC) competencies are invaluable in the care for older adults with complex care needs, but these competencies are not acquired automatically. IntraPC learning requires explicit attention in postgraduate training, where learning usually occurs in the workplace. However, both residents and supervisors struggle with how to incorporate IntraPC learning into daily practice. This study explored how and to what extent current resident-supervisor dialogues (RSDs) provide opportunities for IntraPC learning and explored barriers to IntraPC workplace learning.

**Methods:**

In this focused ethnography, we observed RSDs in the geriatric medicine department of a Dutch university medical center, focusing on opportunities for learning about IntraPC in the workplace. After each observation, both the resident and supervisor participated in in-depth interviews to reflect on the observed behavior. Data were then analyzed through inductive coding followed by thematic analysis. Subsequently, themes were discussed during two focus groups to enable collective reflection with research participants.

**Results:**

Although ample IntraPC learning opportunities were observed in the current RSDs, they remained underutilized. Participants reported dynamics between residents and supervisors that could obstruct IntraPC learning, including perceived time constraints, feedback misalignment between resident and supervisor, monitoring-focus, and the struggle for entrustment.

**Discussion:**

IntraPC learning opportunities in RSDs often remain implicit, even though addressing IntraPC learning does not necessarily require additional supervision time. This implicit learning limits reflection on current collaborative practices. Moreover, dynamics between residents and supervisors may hinder the explicit development of IntraPC competencies. To support improvements in IntraPC learning, supervisors and residents are encouraged to regard IntraPC practices as an important learning topic within RSDs.

## Introduction

The aging society has growing numbers of older patients with multimorbidity and increasing complex care needs [1]. Due to interacting comorbidities, creating personalized treatment plans often requires the perspectives of multiple specialized healthcare professionals [1,2]. A potential risk that arises when multiple healthcare professionals are involved in a single patient’s case, is that they all create their own monodisciplinary treatment plan [3]. Without collaboration, fragmentation of care and conflicting recommendations between the multiple treatment plans may occur [3]. It is therefore important for the various health professionals to learn how to coordinate tasks and align their perspectives to ensure effective collaborative practice [4].

To help ensure future health professionals are prepared to provide effective collaborative practice, medical education research has been aimed at exploring Interprofessional collaboration (IPC) as well as intraprofessional collaboration (IntraPC) [5,6,7]. IPC refers to the process by which healthcare professionals from different professional backgrounds (such as nurses, doctors, and paramedics) collaborate to develop integrated care plans, whereas IntraPC specifically concerns creating an integrated, personalized treatment plan among physicians from different medical specialties (such as surgeons, geriatricians and cardiologists) [8].

While IPC learning has been extensively studied, IntraPC learning remains relatively underexplored [9]. Certain themes within IPC learning such as role clarification and clear communication are also relevant to IntraPC learning. However, there are context-specific aspects of collaboration across medical specialties that may prompt different challenges. These challenges include interactional, hierarchical, and professional identity–related dynamics that impact daily interactions among physicians with different specialties [8,10,11,12]. For example, in interprofessional teams, hierarchical relations are often explicit and tied to professional roles and responsibilities, whereas in intraprofessional teams hierarchy, ownership, responsibility, and task distribution are more implicit and frequently subject to negotiation [8,10,11,12]. These differences highlight the need to conceptualize and study IntraPC learning as a distinct learning phenomenon with its own challenges and learning requirements.

In current postgraduate training, residents primarily learn IntraPC through implicit socialization processes during everyday clinical work [8,11]. This includes both interactions with physicians from other specialties and resident–supervisor dialogues (RSDs) [8,11]. Through participation in routine practice, residents gradually learn the expectations of different specialties and understand how their own role relates to those of others [13]. Research has shown, however, that clinical practice is often characterized by siloed working patterns, and that interactions between physicians from different specialties frequently lack coordination and integration [9]. Relying solely on these implicit learning processes may lead to inconsistent IntraPC learning and perpetuate risks of fragmented care and adverse patient outcomes.

This has led to calls for more explicit attention to IntraPC learning within workplace learning [5,8]. Supervisors play a crucial role in creating a learning environment that facilitates IntraPC workplace learning and resident-supervisor interactions are an important part of workplace learning in postgraduate training [8,11,14]. The key mechanisms of workplace learning during resident-supervisor interactions are mutual observation of practice (monitoring and modelling), supervised participation in action (entrustment and support seeking) and dialogue during practice (meaning making and feedback), also known as RSDs [14] (see also Box 1). [8,11,15].

**Box 1 Contexts and mechanisms of supervised workplace learning as described by Wiese et al [14] Contexts and mechanisms of supervised workplace learning**

**Context: Supervised participation**

The degree to which residents participate in practice

**Table:** 

Entrustment	The supervisor grants the resident the opportunity to perform clinical tasks autonomously, without direct monitoring
Support seeking	The resident seeks the supervisor’s input for a clinical task


**Context: Mutual observation**

Resident and supervisor observe each other’s work

**Table:** 

Monitoring	The supervisor observes the resident’s execution of clinical tasks
Modelling	The resident observes the supervisor’s execution of a clinical task


**Context: Dialogue in practice**

Discussion among resident and supervisor outside of direct patient contact

**Table:** 

Feedback	A mutual co-construction of resident performance and the means to improve it
Meaning making	Shared clinical reasoning through iterative questioning and answering, which aims to stimulate critical thinking and to draw out underlying presumptions

Supervisors and residents rarely observe each other’s collaborative practices, limiting opportunities for learning through direct role-modelling [9,16]. That makes other learning opportunities, such as RSDs, even more important to address IntraPC learning in the workplace [11]. RSDs are moments in which residents and supervisors discuss patient care, reflect on clinical decisions, and collectively make sense of care trajectories [17]. During RSDs, IntraPC learning through shared meaning making may occur as residents may refer to interactions with other specialists, making RSDs a potential site where IntraPC experiences can be surfaced, interpreted, and connected to broader learning goals while remaining embedded in everyday clinical practice [10,18]. It has been argued, that contextual factors such as increasing workload and limited supervision hours form barriers to facilitating IntraPC learning during RSDs or for creating new IntraPC learning opportunities [19]. Additionally, previous research into the supervisors’ perspective on IntraPC workplace learning has indicated that supervisors may question whether IntraPC learning contributes to improved clinical practice [18]. Furthermore, supervisors may feel underprepared to facilitate IntraPC learning [18].

During RSDs, supervisors often struggle to balance interactions focused on overseeing residents’ clinical work with those aimed at reflection and learning, particularly those focused on IntraPC learning [18,20]. As a result, it remains unclear how supervisors and residents, despite contextual barriers, can better utilize existing opportunities for IntraPC learning during RSDs. Given the increasing importance of adequate IntraPC competencies, more knowledge is needed of how supervisors can facilitate IntraPC learning during RSDs. To explore how supervisors might support IntraPC learning despite the complexities of the clinical workplace and the barriers it presents, this study aimed to answer the following research question: How, and to what extent, do existing RSDs provide opportunities for IntraPC learning in the workplace? By exploring current IntraPC learning practices through observations in the workplace we aim to identify how IntraPC workplace learning in RSDs may be improved.

## Methods

We performed a focused ethnography study from a constructivist paradigm [21]. Focused ethnography-based methods provide a means of collecting data within a well-defined timeline and context by using triangulation of observations, interviews and theory [22]. Focused ethnography is an effective method to explore resident-supervisor interactions within a familiar context and limited timeframe [22,23,24]. As focused ethnography requires researchers to be familiar with the context, observations were performed by a medical intern who had done a three month rotation in the studied department prior to data collection. This medical intern was prepared beforehand by senior researchers through trial observations. These senior researchers had different professionals backgrounds, including psychology, educational research and medical specialists, which provided the observer with observational from a broader perspective and increased reflexivity [25].

### Study setting and participants

This study was conducted at the geriatric medicine department at a university medical center in the Netherlands. This geriatric medicine department includes out-patient consultation, an in-patient acute care ward, an attending geriatric resident in the emergency department and a geriatric liaison team for in-patients. Its staff consists of nine geriatricians, two internists and ten residents from different primary and secondary care specialty training programs. Two of the researchers of this study are a part of the staff and were excluded from participation. For several years, IntraPC learning has been a topic of research and resident and supervisor training at this department.

Participants included residents and supervisors working in the geriatric medicine department. Residents were trainee-physicians partaking in a geriatric medicine rotation. The residents’ professional backgrounds including geriatric medicine, internal medicine, general practice and elderly care. Supervisors were geriatricians and internists who oversaw residents’ clinical work and were responsible for supporting workplace learning during RSDs. Further details on how the medical education trajectory is structured in the Netherlands, including how postgraduate training is shaped, can be found in Box 2.

**Box 2 The medical education trajectory and the role of postgraduate residency programs.**

In the Netherlands, the medical education trajectory consists of an undergraduate training that is composed of a bachelor and a master phase followed by postgraduate specialty training. The undergraduate training consists of a combination of acquiring knowledge on health and disease through formal classroom and internships.

After completing undergraduate training, students can either choose to work to gain experience in clinical practice without entering postgraduate training, or they can apply for a specialty postgraduate training program. This specialty training program is mostly composed of workplace learning, where the students become residents and partake in rotations within their own specialty or in out-of-specialty rotations. The progress of their training is overseen by a program director, but their daily work is overseen by supervisors at the workplace, that can change day to day.

The geriatric medicine postgraduate training program lasts five years and includes rotations in internal medicine, cardiology, hospital-based geriatric medicine (including outpatient, emergency and inpatient care), as well as placements in old age psychiatry and neurology.

In the studied geriatric medicine department, not only geriatric medicine residents are present, but also residents from other training programs completing out-of-specialty rotations, including internal medicine, general practice and elderly care. These programs differ in duration and structure. Internal medicine training lasts six years and includes rotations in multiple specialties such as endocrinology, cardiology, rheumatology and geriatric medicine. General practice and elderly care training both last three years and combine rotations in primary care with placements in hospital settings such as emergency medicine, geriatric medicine and psychiatry. During the geriatric medicine rotations in the studied department, residents are divided over the available clinical settings including inpatient acute care, emergency geriatric medicine, outpatients consultation or the geriatric medicine liaison team.

Residents’ daily work is supervised by a clinical supervisor, who is a medical specialist who is responsible for both overseeing their clinical practice and facilitating their workplace learning. Depending on the clinical setting, the supervisor may vary from day to day, ensuring that residents are exposed to different supervisory styles and learning opportunities. This supervision is shaped through various activities, including resident-supervisor dialogues (RSDs), mutual observations, and reflective discussions. As the daily practice often results in a different supervisor every couple of days, a diverse set of supervisors shapes the workplace learning, with the overview of a resident’s growth being overseen by the program director.

Participants were recruited for observations and interviews through purposive sampling. All residents and supervisors were asked to participate in the focus group on a voluntary basis. Two weeks prior to the start of the observations, supervisors and residents were informed about this study through a letter from the secretary of the department asking for their written informed consent. Participants were asked for their written informed consent at the beginning of each interview and focus group. Participants could end observations or interviews at any time, without giving any reason. If stated, the data from observations and/or interviews could be removed from the dataset. Data from focus groups could not be removed, as it would not be possible to identify individual findings within the focus group recordings.

We specifically chose to interview residents and supervisors separately to ensure psychological safety of residents both during in-depth and focus group interviews. As the supervisors jointly have a role in the evaluation of the resident, they otherwise might have felt reservations to give their full opinion on the supervisory practices of their supervisors.

### Data collection

Data collection transpired between October 2023 and January 2024. We started with observations of RSDs which were recorded in observation field notes. Observations of RSDs were performed in all working areas of this department, being out-patient consultation, liaison consultation and the in-patient geriatric acute care ward. Spradley’s nine dimensions of ethnography were used as an observation template [24,26] (Box 3). The observation field notes reported on descriptions, stories, andexperiences that occurred at times the resident and/or supervisor mentioned a ‘third party’ specialty physician or a task, role or responsibility of a different specialty physician. Using the nine dimensions of ethnography, contextual factors during these moments were reported in a narrative manner. Interpretations were written in a different color, so it was clear which observations could contain the observer’s perspectives or underlying beliefs [24].

**Box 3 Nine dimensions of ethnography by Spradley (26)**

**Table:** 

Dimension	Descriptor
Space	Physical layout of the place(s)
Actor	Range of people involved
Activity	A set of related activities that occur
Object	The physical things that are present
Act	Single actions people undertake
Event	Activities that people carry out
Time	The sequencing of events that occur
Goal	Things that people are trying to accomplish
Feeling	Emotions felt and expressed

In the course of these observations, we identified opportunities for IntraPC learning, which we defined as instances and cues in clinical practice where tasks or activities involving a physician from another specialty were referenced, potentially leading to discussions that could involve IntraPC learning.

Observations were followed by in-depth interviews with the observed residents and the supervisors separately. The interview guide consisted of a basic set of questions to discuss general ideas of the participant on clinical supervision and the observed RSDs. The interview guide was supplemented with specific observations of that participant, to explore whether the participant had experienced IntraPC learning moments and what outcomes these yielded, to explore observer interpretations or to explore underlying motives and beliefs behind observed behavior. As previous work has shown that residents and supervisors may be unable to identify IntraPC learning opportunities, the researchers identified moments with potential for IntraPC learning during observations and discussed these during interviews when participants did not mention any IntraPC learning opportunities [8]. This was done to explore additional barriers to IntraPC learning beyond the recognition of everyday IntraPC learning opportunities during the interviews.

To formulate these explorative questions we drew on insights from reflective interviewing, rather than using a strict theoretical framework [27]. This entailed focusing on an open and inquiry-based approach, active listening, and reflection on underlying assumptions [27].

The first interviews were performed by two researchers to ensure consistency and to train the medical intern. The interviews started with exploring their experiences of the observed dialogues supplemented with questions to clarify observed behavior. Interviews were then transcribed verbatim.

Observations and interviews continued until the data analysis indicated that data sufficiency and theoretical sufficiency had been reached and no new valuable codes were added to the code book [28]. After data analysis of the observations and interviews had been completed, one separate focus group with residents and one separate with supervisors was held. These focus groups were performed by two researchers with different backgrounds (medical intern IW and psychologist NL) to include multiple perspectives during data collection. (Appendix: interview guides)

### Data analysis

Observation fieldnotes and interview transcripts were analyzed through inductive coding in Atlas.ti. Two researchers (IW and MV) assessed each field note and transcript individually using open coding. IW and MV met weekly to discuss discrepancies in codes and to create one code book. The code book and relevant quotes were discussed during monthly meeting with the entire research group. During these discussions, the diverse perspectives within the research group were brought into the data analysis, providing for a rigorous analysis and increased reflexivity [25].

Once the code book was complete, two researchers performed thematic analysis through analytic memoing to categorize data into preliminary themes [29]. The preliminary themes were discussed with the research group, who provided theoretical insight. During the meetings we identified the dynamics between residents and supervisors that impacted IntraPC learning.

To check these preliminary themes with study participants and to gain a deeper understanding of the resident-supervisor dynamics, we performed focus groups to enable collective reflection with residents and supervisors. We conducted one separate focus group with residents and one separate with supervisors to create a safe environment for both to speak freely.

**Figure 1:**
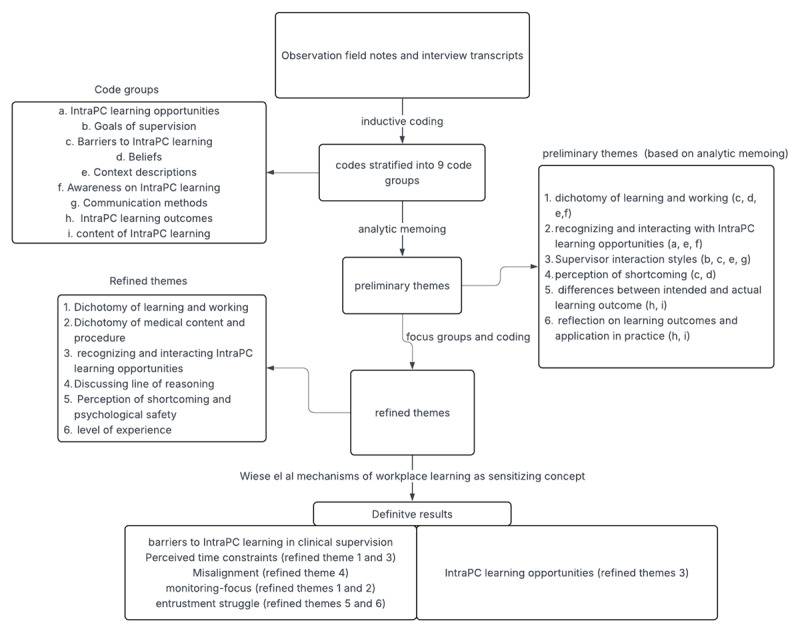
Process of data analysis.

The focus group interviews were transcribed verbatim and analyzed with the same strategy as the observations and interviews. The mechanisms of supervised workplace learning as described by Wiese et al. were used as sensitizing concepts to further understand the refined themes [14]. Finally, data from observations, interviews and focus groups were then triangulated into definitive results [28] (Figure 1).

### Ethical approval

Ethical approval was provided by the NVMO Ethical Review Board (NERB dossier number 2023.5.4).

## Results

In total, we performed 43 observations (lasting 6 to 142 minutes per observation) with a total observation time of 22 hours: 20 observations were performed in outpatient consultation, 12 observations in liaison consultation and 11 observations in the inpatient ward. The longest observations (20A and 20B) were during a shared ward round with both the supervisor and the resident in the inpatient ward.

Purposive sampling resulted in the inclusion of all 9 non-researcher supervisors and 7 residents from different specialty training programs (Table 1). All observed residents and supervisors agreed to participate in interviews, resulting in a total of 19 interviews, including 10 residents and 9 supervisors. Interviews lasted between 17 and 55 minutes. The two separate focus groups consisted of 4 residents and 9 supervisors respectively. Interview and focus group participants’ characteristics are presented in Table 1. All supervisors participated in the focus group and 4 residents out of the 10 residents agreed to participate in the focus group. To maximize potential participation for the focus group, a regular teaching hour was used. Evening and night shift scheduling with subsequent resting hours were the most common reasons for residents to refrain from participation.

**Table 1:** Interview participants’ characteristics.


	OBSERVATIONS AND INTERVIEWS	FOCUS GROUPS

Residents		

**Age (range)**	24–35	24–35

**Sex (F/M)**	7/0	4/0

**Years of experience (range)**	1–5	1–5

**Specialty (N)**		

Primary care		

General Practice	2	0

Nursing home care	1	1

Secondary care		

Geriatric Medicine	5	2

Internal Medicine	2	1

Supervisors		

**Age (range)**	35–67	35–67

**Sex (F/M)**	6/3	6/3

**Specialty**		

Geriatric Medicine	7	7

Internal Medicine	2	2


We observed that many RSDs contained potential IntraPC learning opportunities.

These learning opportunities occurred when residents and supervisors:

– **conveyed a (non-verbal) emotion about an interaction with another physician;**
  These emotions varied, including signs of either positive or negative reactions towards the practices of physicians from other specialties. In some instances, these emotional responses seemed to reflect underlying beliefs or perceptions, which could indicate the presence of differing views, prejudice or power dynamics.
– **discussed their specialty’s role and responsibilities within the patient’s wider care network;**
  These discussions included how they regarded their roles, tasks and responsibilities and how others may have different perspectives towards these elements in clinical practice
– **discussed a task for another specialty physician as part of collaborative care**.
  These discussions included whether they formulated why the consultation was necessary, what they expected from this specialty physician and what this consultation might mean and how they perceived the consultation’s potential contribution to the patient’s care.

Despite the presence of learning opportunities, residents and supervisors did not always explicate the IntraPC learning or collaborative practices during these discussions.

During our analysis of potential barriers to IntraPC learning in the RSDs, we identified the following themes: perceived time constraints, feedback misalignment, monitoring-focus and entrustment struggle. Our findings on learning opportunities and barriers to IntraPC learning in RSDs are presented below and are summarized in Table 2 in Appendix 2.

### Perceived time constraints

As we discussed learning opportunities that had remained unused, two key reactions emerged: either the opportunity was not recognized by the supervisor or the resident, or it was not effectively utilized for learning due to time constraints and competing priorities.

*“And anything that I’m feeling sort of insecure about or that is about communication, these are often things that can wait.”* – Resident focus group

Time constraints were mentioned as the most pressing barrier to reflecting on these learning opportunities. In this geriatric medicine department, the agreed duration of an individual RSD was approximately 30 minutes. However, our observations revealed considerable variation in the duration of RSDs, depending on the clinical context and situational factors.

Longer RSDs were typically observed when particularly complex care needs arose, when multiple interruptions occurred, or when supervisors deliberately created space for the intern’s knowledge acquisition. In contrast, shorter RSDs were often characterized by supervisors appearing rushed, reflecting competing clinical responsibilities.

Despite this variation in duration, comparing RSDs with and without IntraPC learning moments across different work contexts (outpatient consultations, inpatient acute care, and the liaison team) revealed no clear difference in length (Table 3, Appendix 3). This suggests that explicitly addressing IntraPC learning opportunities does not necessarily require additional supervision time.

### Feedback misalignment

We observed that supervisors provided feedback to residents during RSDs, but the aim of the feedback often remained implicit. In the interviews, we engaged with both supervisors and residents to explore their interpretations of the observed feedback. These interviews contained discrepancies between the supervisors’ intentions and the residents’ interpretations of the feedback. Whereas the supervisor wanted to address an IntraPC topic, the residents reported entirely different learning outcomes based on the supervisor’s feedback than what the supervisor had intended, pertaining to medical knowledge acquisition or doctor-patient communication over IntraPC learning.

An example of misaligning feedback intention and interpretation was when we observed how a supervisor provided the resident with feedback on a referral letter. The quotes below reflect what the supervisor’s feedback intention was, as well as what the resident had interpreted as a learning outcome from this feedback.

*“… and what I felt was important is that I’d heard things behind the scenes that were not mentioned in that letter, which would affect the expectations of the subsequent healthcare professional but which were not officially communicated at all. This bothered me, so I felt it was important that if you’re sending a letter, it should contain everything that matters.”* – Supervisor on feedback intentions

*‘’Patients can read everything these days. So you need to think very carefully about your wording.”* – Resident on interpretation of feedback

When reflecting on misalignment in a supervisor’s intention and a resident’s interpretation during interviews, residents and supervisors mentioned that learning during RSDs is mostly implicit. A supervisor mentioned that it is common for learning outcomes from RSDs to be implicit and that, therefore, RSD learning outcomes rarely lead to adjustments in clinical practice.

*“I don’t think the goal of some feedback is always made explicit.”* – Resident focus group

*“I do think that these are more or less subconscious lessons that you take away from it as a kind of experience. But I don’t think they are very explicit and clear learning points in the sense of: well, now we’ve learned this or that, or: this needs to be documented, or: we need to adjust our protocol.”* – Supervisor interview

When asked why the feedback intention in RSDs often remained implicit, supervisors agreed that being explicit about feedback intention and/or interpretation could hamper psychological safety.

*“This happens occasionally for if I were to keep asking ‘so what is it that you’ve learned’ I guess things would soon begin to feel a little unsafe for them.”* – Supervisor focus group

### Motoring-focus

In our observations, residents usually spent the majority of the dialogue presenting their findings and discussing diagnostic or treatment plans, so the supervisors could monitor if these plans were adequate for this patient. Interactions aimed at meaning making, by reflecting on current IntraPC behavior, for example, where less frequent. During interviews, residents and supervisors explained that they felt RSDs were primarily meant for checking their specialty’s diagnostic and treatment plans. They felt that learning through reflection and IntraPC learning during RSDs was only a secondary goal.

*“No, it’s not a learning opportunity in that case, ‘cause the focus is entirely on checking practical items off the list, tick, tick, tick, until we’re done fire-fighting, let’s move on.”* – Resident interview

*“ Yes, the main goal is of course that I’m trying to establish what’s the matter with the patient or whether the resident’s idea about this patient is right. Then we might see if there’s any learning point, or I might think well, it ‘d be nice to tell them a little more about this and turn it into an educational moment.”* – Supervisor interview

In the supervisor focus group, time constraints were mentioned as barriers to facilitating reflection, causing them to prioritize medical technical discussions over facilitating IntraPC learning.

*“All patients must be discussed, briefly, in main points and above all content-related, so it’s not necessarily about my own learning process.”* – Resident focus group

*“If there’s a lot of medical stuff to be discussed or figured out, well, these kinds of in-depth questions or interprofessional learning issues tend to come second, perhaps.”* – Supervisor focus group

In the focus group, residents underscored that work processes were prioritized over learning processes as the swift execution of clinical tasks was their primary task. They agreed that the primary tasks of interns are learning, but for residents it is effectively performing all necessary tasks in patient care.

*‘’I: I also heard you say: “The resident is really here to learn.” So why are you [residents] here?*

*R: We are the slaves of the ward. […] Because the work needs to be done.”* – Resident focus group

On the other hand, the residents’ mindset on prioritizing monitoring over meaning making, did also obstruct learning when a supervisor would initiate meaning making discussions during the RSDs. Residents mentioned that RSDs were meant to seek the supervisors’ blessing and check whether they had the “correct” treatment plan. When residents felt that a medical technical topic had been underdiscussed during the RSD, they could feel uneasy about whether they had created an adequate treatment plan.

*“Because you need to proceed with your patient that day. So you need to perform tasks that require the approval or blessing of the person supervising you.”* – Resident focus group

Residents would feel frustrated when a focus on IntraPC learning came too early in the RSD, as they would still be too pre-occupied with the creation of a suitable treatment plan for the patient to partake in reflection.

“*Even a little annoyed perhaps ‘cause you’re thinking: come on, I’d first like to know what I’m supposed to do with my furosemide please.”* – Resident focus group

### Entrustment struggle

During the observed RSDs, differences in a resident’s proposed treatment plan and the supervisor’s proposed treatment plan elicited nonverbal responses in residents.

When exploring these responses during interviews, we discovered that residents were constantly seeking approval of their capabilities as a physician. This could result in feelings of personal failure when their supervisor had other viewpoints on treatment plans or asked multiple clarifying questions. Residents reported that they felt a sense of unease when supervisors asked them multiple questions, as this could be interpreted as limited entrustment. RSDs containing multiple questions, therefore, even if these were aimed at meaning making instead of monitoring, could create a tense learning environment and could obstruct IntraPC learning.

*“And if they do so, and then another question, and then another one, then the conversation tends to lose its flow and turns into a kind of exam. I find such communication uncomfortable because it affects my sense of safety.”* – Resident interview

During supervisor interviews, supervisors explained that they were constantly deliberating when to provide entrustment. Entrustment could be provided by refraining from asking monitoring questions on specific entrusted clinical tasks. When no monitoring questions were asked, residents were responsible for the quality of this task. Supervisors repeatedly mentioned that they aimed to adjust their supervision to the residents’ level of experience and their entrusted activities and that they would address other learning goals based on previously entrusted professional activities.

*“Well, what I find particularly important is that you try to match the resident’s level. We have quite a range of levels of experience here, of course, and I really make an effort to appraise: who is this person in front of me and what would be a learning opportunity for him or her*?” – Supervisor interview

Supervisors struggled to balance entrustment with exploring IntraPC learning opportunities. When supervising less experienced residents with fewer entrusted activities, supervisors focused RSDs on the medical technical aspects of clinical practice, and IntraPC was considered to be less urgent and too complex for these residents. With the more experienced residents, who had more entrusted medical technical skills, supervisors felt that RSDs would provide more time for discussion of metacognitive aspects such as reflection on IntraPC. More experienced residents themselves, however, felt that IntraPC was a basic skill that would not require dedicated attention in RSDs of residents further along in their training, where they felt less of a need to address IntraPC in RSDs.

“*That, of course, is quite another level of supervision. […] I feel that such collaboration, these more in-depth effectiveness issues, true collaboration, I’ll keep that for a slightly later stage*.” – Supervisor interview

*“I think it’s part and parcel of this stage of my training that I make and have a plan of my own. And that we should collaborate in this way to deal with something in mutual consultation and a pleasant atmosphere.”* – Resident interview

During the focus group, residents reflected on these findings by saying that when they are aware that the supervisors’ questioning was aimed at facilitating workplace learning, they would feel more at ease during the RSD. In the supervisors’ focus group, some supervisors were surprised by the fact that extensive questioning could impact resident’s safety. Other supervisors agreed that the resident’s mindset upon entering the dialogue could affect their response: residents with a reflective attitude, might feel safer to learn during RSDs.

*“This is no surprise to me. I really get it that this would be your interpretation as a resident. What’s involved here, I think, is the safety you’re offering in your communication with residents. They might feel (as a kind of fixed mindset, you know): ‘Oh dear, I’ve made a mistake and I need to do this differently.’ Or: ‘I’m allowed to develop and the questions will help me to make progress together.’ I hope they feel the latter, but I can readily imagine that supervisors interpret this differently than residents.”* – Supervisor focus group

## Discussion

Through this ethnographic study we aimed to explore whether and how existing RSDs provide opportunities for learning about IntraPC and how this learning could be improved during RSDs. Our findings show that daily RSDs in the care of older adults contain IntraPC learning opportunities which remain mostly unused. These opportunities occur when a (non-verbal) emotion about another specialty physician is conveyed, when residents and supervisors discuss their specialty’s tasks and roles in a broader patient care network or when a new task is assigned to another specialty. IntraPC learning appeared to be influenced by perceived time constraints, feedback misalignment between supervisor and resident, monitoring-focus and the entrustment struggle.

In the care for older adults with complex care needs, coordinated and integrated care is essential to prevent adverse outcomes [1,30]. Current collaborative practices often lack the coordination and integration these patients’ complex care needs demand [9]. In a context where coordination and integration across specialties is lacking, IntraPC learning cannot be expected to be learned implicitly. Moreover, by leaving IntraPC learning implicit during workplace learning, residents may inadvertently acquire undesirable habits (such as a silo mentality) or reinforce power dynamics and prejudice [9,31]. This suggests that adjustments are needed within existing RSDs to better support IntraPC learning.

Within these RSDs, we demonstrated how dynamics between resident and supervisor in supervised workplace learning occur and impact IntraPC learning. In this study, we specified *where* IntraPC opportunities arise within everyday RSDs and *why* they remain unused as a result of these dynamics. Creating treatment plans and acquiring medical knowledge tend to be prioritized during RSDs, resulting in IntraPC learning being systematically deprioritized. Although it is neither feasible nor desirable to make all aspects of practice explicit an individual RSD, current RSDs often treat clinical decision-making (“what to do”) and the collaborative processes required to achieve this (“how to do it together”) as separate aspects of care. In complex care for frail older adults, however, both aspects are essential for good clinical practice.

Our study shows that hierarchical power dynamics appear to limit the use of opportunities for IntraPC Learning in RSDs. The residents’ quotes reveal their hierarchical and evaluative perception of RSDs, suggesting that adjustments to these sessions are unlikely to be initiated by residents themselves. They describe primarily using RSDs to seek validation from supervisors and experiencing their work as service-oriented, referring to themselves as ‘slaves to the ward.’ These dynamics foster a learning environment where there is no clear or optimal opportunity to report on intraprofessional interactions or to reflect on and learn from these experiences. Supervisors may perceive IntraPC learning as too demanding for less experienced residents, while more experienced residents might consider collaboration as a ‘basic skill’ which they should not ‘bother their boss’ with. As a result, residents may avoid raising collaborative challenges for fear of appearing incompetent or vulnerable in evaluative situations with their supervisors. Consequently, residents rarely report on interactions with physicians from other specialties unless overt conflicts arise in clinical practice.

These findings build on previous research, which shows that implicit biases between specialties can influence interactions between residents, and how power dynamics may lead to forms of intraprofessional silencing, whereby the perspectives of certain specialties are lost in communication between residents and supervisors [31,32]. These mechanisms further limit opportunities for supervisors to gain insight into how collaboration between specialties unfolds.

Our findings suggest that supervisors are key to fostering more explicit IntraPC learning. Importantly, supervisors can make a meaningful difference through relatively small adjustments to existing RSDs. Insights from IPC research show that supervisors can prime residents to learn from everyday interactions by encouraging reflection-in-action and by making power dynamics in collaborative encounters explicit [33]. What this study adds is the need to extend such attention beyond IPC to IntraPC, as IntraPC involves different hierarchies and power dynamics, which otherwise can easily be overlooked and taken for granted. Supervisors need to recognize IntraPC as an integral component of good clinical practice and to monitor it with the same attentiveness as creating diagnostic and treatment plans.

A first step is for supervisors to inquire more actively into how collaboration across specialties unfolded or will be addressed. Supervisors are often not directly involved in these interactions as they are often performing other clinical, scientific or educational responsibilities simultaneously, while residents do not spontaneously report on collaborative practice [19]. By asking exploratory, process-oriented questions, supervisors can create space for residents to reflect on collaborative practices. This requires supervisors to view care for patients with complex needs as part of a broader care continuum, attending not only to what happens within their own specialty, but also to what precedes and follows that encounter. Actively inviting residents to reflect on these connections encourages them to consider how care across specialties is aligned and coordinated.

Supervisors may initially perceive such adjustments as unnecessary [9,18]. Other remarks include that such adjustments might require large-scale cultural change through extensive training programs, which supervisors oppose due to competing priorities [9,18]. Large-scale cultural adjustments are known to be difficult to implement in complex clinical environments, as clinical activities and learning activities in post-graduate training are already extensive [34,35]. However, our findings suggest that improvements for IntraPC may not require complex large-scale training programs. Precisely because collaboration across specialties is so prevalent in everyday practice, supervisors can stimulate IntraPC learning by modestly broadening their monitoring practices to include not only the diagnostic and treatment plan, but also how collaboration across specialties is or can be organized. This requires supervisors to recognize coordination and integration of care as integral components of good clinical practice and to validate their discussion during RSDs, thereby extending their role beyond evaluating clinical decisions to also guiding residents in the collaborative practice across specialties. In doing so, supervisors can support residents in developing IntraPC competencies which are required for more coordinated and integrated care for older adults with complex care needs.

### Implications

This study’s observed IntraPC learning opportunities suggest that IntraPC learning can be meaningfully supported within existing RSDs through small, context-sensitive adjustments rather than through extensive training programs. Supervisors play a key role by modestly broadening their monitoring focus during routine RSDs to include both clinical decision-making and how care is coordinated across medical specialties.

Supervisors can embed brief, process-oriented questions into everyday supervisory conversations, such as: “How shall we align with the care provided by the other specialist?” “What do we need from other specialties, and what do they need from us?” and “How did the collaboration with the other specialty go?” Through these prompts, supervisors can make collaborative processes visible and establish them as legitimate learning objectives. Such prompts signal how care is organised across specialties is as clinically relevant as what decisions are made. By consistently attending to collaborative processes across the care continuum, supervisors can support residents in developing the competencies needed for coordinated and integrated care, particularly for older adults with complex health needs.

This study also illustrates the value of ethnographic approaches for uncovering how learning opportunities emerge and disappear in real-time clinical interactions. Future research could build on these findings by examining how brief, process-oriented supervisory prompts influence residents’ collaborative practices over time and how such practices affect care coordination and patient outcomes. Longitudinal and intervention-based studies may further elucidate whether making IntraPC explicit within supervision contributes to more coordinated and integrated care.

### Strengths and limitations

This study has several strengths. Firstly, we incorporated the perspective of both residents and supervisors on IntraPC workplace learning, whereas previous studies typically focused on only one of these two perspectives. Additionally, by using combined multiple methods of data collection – observations, interviews and focus groups – we could triangulate our findings and report on both observed and reported IntraPC learning behavior. The use of focus groups was a particular strength of this study, as they created an opportunity for collective reflection on the data with both researchers and research participants. This approach together with the discussions with the research team which represented diverse perspectives, contributed to a rigorous and reflexive data analysis.

Our study also had some limitations. The study was conducted within a single center, whereas individual findings may be different in other clinical settings. Most large medical centers, however, share similar complex dynamics. These dynamics may also play a role in the learning of other metacognitive competencies such as interprofessional collaboration or medical ethical aspects of a patient’s case. We believe, therefore, that the results are transferable to a variety of other clinical settings.

Furthermore, the study took place within a single department where attention had already been given to IntraPC learning. Yet the findings indicate that even in this setting with a considerable pre-existing focus on IntraPC learning, IntraPC learning potential in RSDs for IntraPC remains under exploited. This underscores the importance of addressing IntraPC learning across diverse clinical settings involved in complex medical care, specifically through targeted adjustments in RSDs, which can facilitate more explicit communication about IntraPC learning.

## Conclusion

Resident-supervisor dialogues (RSDs) offer ample opportunities to facilitate learning about intraprofessional collaboration (IntraPC), which when explored, do not necessarily increase the duration of RSDs. Although implicit IntraPC learning does occur, opportunities to reflect on and transform current collaborative practice remain unused. The dynamics between resident and supervisor may obstruct IntraPC learning, including perceived time constraints, feedback misalignment, monitoring-focus and the entrustment struggle. In order to improve IntraPC learning, supervisors and residents need to become aware of how procedural aspects of care are vital to provide good clinical practice and would benefit from small context-sensitive adjustments to clinical supervision to create space for discussion on IntraPC during RSDs.

## Additional Files

The additional files for this article can be found as follows:

10.5334/pme.1863.s1 Appendix 1. Interview guides.

10.5334/pme.1863.s2 Appendix 2. Table 2.

10.5334/pme.1863.s3 Appendix 3. Table 3.

## Abbreviations

IntraPC: intraprofessional collaboration.

RSDs: resident-supervisor dialogues.

## References

[1] Pefoyo AJ, Bronskill SE, Gruneir A, Calzavara A, Thavorn K, Petrosyan Y, et al. The increasing burden and complexity of multimorbidity. BMC Public Health. 2015;15:415. DOI: 10.1186/s12889-015-1733-2 25903064 PMC4415224

[2] Bajeux E, Corvol A, Somme D. Integrated Care for Older People in France in 2020: Findings, Challenges, and Prospects. Int J Integr Care. 2021;21(4):16. DOI: 10.5334/ijic.5643 PMC8588900 34824565

[3] Bakewell F. Medical silos, social identity, and duty of care: A call for health leaders to improve transitions of care. Healthc Manage Forum. 2024:8404704241290689. DOI: 10.1177/08404704241290689 PMC11849241 39425269

[4] Meijer LJ, de Groot E, Honing-de Lange G, Kearney G, Schellevis FG, Damoiseaux R. Transcending boundaries for collaborative patient care. Med Teach. 2021;43(1):27–31. DOI: 10.1080/0142159X.2020.1796947 32767903

[5] Looman N, de Graaf J, Thoonen B, van Asselt D, de Groot E, Kramer A, et al. Designing the learning of intraprofessional collaboration among medical residents. Med Educ. 2022;56(10):1017–1031. DOI: 10.1111/medu.14868 35791303 PMC9543842

[6] Clark PG. Narrative in interprofessional education and practice: implications for professional identity, provider-patient communication and teamwork. J Interprof Care. 2014;28(1):34–39. DOI: 10.3109/13561820.2013.853652 24224865

[7] de Nooijer J, Dolmans DHJM, Stalmeijer RE. Applying Landscapes of Practice Principles to the Design of Interprofessional Education. Teach Learn Med. 2022;34(2):209–214. DOI: 10.1080/10401334.2021.1904937 33789558

[8] Teheux L, Coolen E, Draaisma JMT, de Visser M, Scherpbier-de Haan ND, Kuijer-Siebelink W, et al. Intraprofessional workplace learning in postgraduate medical education: a scoping review. BMC Med Educ. 2021;21(1):479. DOI: 10.1186/s12909-021-02910-6 34493263 PMC8424991

[9] van der Ven M, Looman N, Ergun- Al Kafadji N, Dalloyaux S, Sir O, Braspenning J, et al. The impact of context on interphysician collaboration and learning: A focused ethnography around hip fracture patients in the emergency department. SSM – Qualitative Research in Health. 2025;7:100566. DOI: 10.1016/j.ssmqr.2025.100566

[10] Teheux L, van der Velden J. Untapped opportunities: Leveraging the entire health care team in workplace learning. Med Educ. 2025;59(5):457–459. DOI: 10.1111/medu.15618 39902630 PMC11976207

[11] Looman N, Fluit C, van Wijngaarden M, de Groot E, Dielissen P, van Asselt D, et al. Chances for learning intraprofessional collaboration between residents in hospitals. Med Educ. 2020;54(12):1109–1119. DOI: 10.1111/medu.14279 32564390 PMC7754101

[12] Krystallidou D, Kersbergen MJ, de Groot E, Fluit C, Kuijer-Siebelink W, Mertens F, et al. Interprofessional education for healthcare professionals. A BEME realist review of what works, why, for whom and in what circumstances in undergraduate health sciences education: BEME Guide No. 83. Med Teach. 2024;46(12):1607–1624. DOI: 10.1080/0142159X.2024.2314203 38513054

[13] Galema G, Duvivier R, Pols J, Jaarsma D, Wietasch G. Learning the ropes: strategies program directors use to facilitate organizational socialization of newcomer residents, a qualitative study. BMC Med Educ. 2022;22(1):247. DOI: 10.1186/s12909-022-03315-9 35382804 PMC8981951

[14] Wiese A, Kilty C, Bennett D. Supervised workplace learning in postgraduate training: a realist synthesis. Med Educ. 2018;52(9):951–969. DOI: 10.1111/medu.13655

[15] Teheux L, Kuijer-Siebelink W, Bus LL, Draaisma JMT, Coolen EHAJ, van der Velden JAEM. Unravelling underlying processes in intraprofessional workplace learning in residency. Med Educ.n/a(n/a). 10.1111/medu.15271 37990961

[16] Hazen ACM, de Groot E, de Bont AA, de Vocht S, de Gier JJ, Bouvy ML, et al. Learning Through Boundary Crossing: Professional Identity Formation of Pharmacists Transitioning to General Practice in the Netherlands. Academic Medicine. 2018;93(10):1531–1538. DOI: 10.1097/ACM.0000000000002180 29465448

[17] Gamborg ML, Jensen RD, Musaeus P, Mylopoulos M. Balancing closure and discovery: adaptive expertise in the workplace. Adv Health Sci Educ Theory Pract. 2022;27(5):1317–1330. DOI: 10.1007/s10459-022-10177-9 36418756

[18] de Groot E, van den Broek M, Fokkens JT, Witte JAM, Damoiseaux R, Zwart DLM. Supervisors’ pedagogies for supporting interns to learn intra- and interprofessional collaboration: a qualitative and quantitative ego network analysis. J Interprof Care. 2021;35(2):185–192. DOI: 10.1080/13561820.2020.1712336 32037921

[19] Banerjee G, Mitchell JD, Brzezinski M, DePorre A, Ballard HA. Burnout in Academic Physicians. Perm J. 2023;27(2):142–149. DOI: 10.7812/TPP/23.032 37309180 PMC10266848

[20] Robbrecht M, Van Winckel M, Norga K, Embo M. Exploring residents and supervisors’ workplace learning needs during postgraduate medical education. Int J Med Educ. 2023;14:65–74. DOI: 10.5116/ijme.6470.d9ed 37269308 PMC10693396

[21] Ng SL, Kinsella EA, Friesen F, Hodges B. Reclaiming a theoretical orientation to reflection in medical education research: a critical narrative review. Med Educ. 2015;49(5):461–475. DOI: 10.1111/medu.12680 25924122

[22] Andreassen P, Christensen MK, Møller JE. Focused ethnography as an approach in medical education research. Med Educ. 2020;54(4):296–302. DOI: 10.1111/medu.14045 31850537

[23] Berkhout JJ, Helmich E, Tuenissen PW. The complex relationship between student, context and learning outcomes. Med Educ. 2016;50(2):164–166. DOI: 10.1111/medu.12950 26812994

[24] Reeves S, Peller J, Goldman J, Kitto S. Ethnography in qualitative educational research: AMEE Guide No. 80. Med Teach. 2013;35(8):e1365–e1379. DOI: 10.3109/0142159X.2013.804977 23808715

[25] Barry CA, Britten N, Barber N, Bradley C, Stevenson F. Using reflexivity to optimize teamwork in qualitative research. Qual Health Res. 1999;9(1):26–44. DOI: 10.1177/104973299129121677 10558357

[26] Spradley J. Interviewing in ethnology: New York : Holt, Rinehart and Winston; 1979.

[27] Roulston K. Reflective Interviewing: A Guide to Theory and Practice. London: SAGE Publications Ltd; 2010. Available from: https://methods.sagepub.com/book/mono/reflective-interviewing/toc.

[28] Varpio L, Ajjawi R, Monrouxe LV, O’Brien BC, Rees CE. Shedding the cobra effect: problematising thematic emergence, triangulation, saturation and member checking. Med Educ. 2017;51(1):40–50. DOI: 10.1111/medu.13124 27981658

[29] Kiger ME, Varpio L. Thematic analysis of qualitative data: AMEE Guide No. 131. Med Teach. 2020;42(8):846–854. DOI: 10.1080/0142159X.2020.1755030 32356468

[30] Barnett K, Mercer SW, Norbury M, Watt G, Wyke S, Guthrie B. Epidemiology of multimorbidity and implications for health care, research, and medical education: a cross-sectional study. Lancet. 2012;380(9836):37–43. DOI: 10.1016/S0140-6736(12)60240-2 22579043

[31] Looman N, van Woezik T, van Asselt D, Scherpbier-de Haan N, Fluit C, de Graaf J. Exploring power dynamics and their impact on intraprofessional learning. Med Educ. 2022;56(4):444–455. DOI: 10.1111/medu.14706 34841565 PMC9300127

[32] Looman N, Battal N, Vanstone M. No doctor is an island. Med Educ. 2023;57(11):996–998. DOI: 10.1111/medu.15168 37490936

[33] Miller KA, Ilgen JS, de Bruin ABH, Pusic MV, Stalmeijer RE. Physician development through interprofessional workplace interactions: A critical review. Med Educ. 2025;59(5):484–493. DOI: 10.1111/medu.15564 39440879

[34] Gupta S, Higgins S, Torre D. Wellbeing and Burnout in Residency. J Gen Intern Med. 2022;37(9):2137–2138. DOI: 10.1007/s11606-022-07663-6 35606642 PMC9126691

[35] Teunissen PW. Experience, trajectories, and reifications: an emerging framework of practice-based learning in healthcare workplaces. Adv Health Sci Educ Theory Pract. 2015;20(4):843–856. DOI: 10.1007/s10459-014-9556-y 25269765

