# Supplementary material for: An Offer You Can’t Refuse: Opportunities for Intraprofessional Collaboration Learning in the Workplace
Source: Perspect Med Educ. 2026 Jul 13;15(1):586–99. doi: 10.5334/pme.1863 (PMC13378418; doi:10.5334/pme.1863)
Supplement: Appendix 1. — Interview guides. [file pme-15-1-1863-s1.pdf]

Appendix 1: interview guides

interview guide in-depth interview

Thank you for agreeing to participate in this interview. Before we begin, do you consent to the interview being recorded and used for scientific research?

This study aims to investigate how interphysician collaboration learning opportunities may be utilized for workplace learning in the interactions between residents and supervisors. We conducted observations, followed by this interviews to clarify certain aspects of what was observed. There are no right or wrong answers—we are interested in your perspective. Do you have any questions before we start?

Alright, first question: How do you look back on the observed supervision moments?

What was your goal for these supervision sessions?

What is important to you in supervision?

What role does learning interphysician collaboration play in that?

I'd like to discuss a few notable observations with you: [insert observations]

Do you recognize this moment?

How did you experience that moment?

What did you learn from it?

What did you learn about interphysician collaboration from it?

[if needed, insert more observations]

Interview guide: focus group

Good afternoon everyone, and welcome to this focus group for the study on interphysician collaboration learning. In this study we would like to explore with you what opportunities exist for learning interphysician collaboration during workplace learning, and how these opportunities can be better utilized.

Do you agree to participate, with your data being used pseudonymously for a scientific publication? To do so, this session will be recorded—do you also agree to that? If so, could you please sign the informed consent form and hand it in to me?

In this focus group, we are curious about your perspective on the data collected from the observations and interviews. There are no right or wrong answers—we are primarily interested in your point of view. Are there any questions before we begin?

First, the observing researcher will provide an overview of the results.
We conducted 44 observations in which we mapped the learning process during supervision. From this, we identified six themes that complicate intraprofessional learning. These are (see table).

Now that you've heard these themes, what comes to mind? *Follow-up probing questions:*
Do you recognize these themes? Why or why not?
How do these themes affect you?
What do these themes bring to mind for you?
What could we do to improve collaborative learning, keeping these themes in mind?
What would be needed for that?

Dichotomy of Learning and Working	Learning and working are perceived as two separate processes that (often) cannot occur simultaneously.
Recognizing and Interacting with Learning Opportunities	There are many opportunities for learning intraprofessional collaboration, but these moments do not always seem to be recognized or utilized.<br>Examples include:<br>A judgment about another specialist is implied through tone of voice or a change in atmosphere by the resident.<br>The resident discusses another specialist's request for help.<br>The supervisor gives the resident the task of contacting another healthcare provider
Supervisor Interaction Style	We observed three interaction styles among supervisors:<br>**Passive**: supervision based on questions posed by the resident<br>**Reactive**: the resident takes the lead, while the supervisor probes with follow-up questions<br>**Proactive**: the supervisor takes the lead and shares personal experiences<br>Behavior may vary between individuals and depending on the topic of conversation.<br>Proactive interaction appears to more often lead to learning moments about collaboration.

Perception of Shortcomings by Residents	When learning moments are created during supervision, residents may feel that they are “not good enough” as doctors. This feeling appears to increase the longer the supervision lasts.
Difference Between Intended and Actual Learning Outcomes	The supervisor creates a learning moment about collaboration, but the resident may take away a completely different lesson.
Reflection on Learning and Application in Practice	Even when learning about collaboration occurs, there is not always reflection on how this learning can be translated into new practices.
