# Supplementary material for: An Offer You Can’t Refuse: Opportunities for Intraprofessional Collaboration Learning in the Workplace
Source: Perspect Med Educ. 2026 Jul 13;15(1):586–99. doi: 10.5334/pme.1863 (PMC13378418; doi:10.5334/pme.1863)
Supplement: Appendix 2. — Table 2. [file pme-15-1-1863-s2.pdf]

Appendix 2

Table 2: summary of results on IntraPC learning opportunities and barriers to IntraPC learning

**IntraPC learning opportunities**
**When resident and/or supervisor:**<br>**1. convey a (non verbal) emotion about an interaction with another physician**<br>'At that [specialty] department they are not that good at [geriatric] care, so we will have to pay extra attention over there' – field note observation liaison consultation team<br><br>'Sometimes we receive referrals from general practitioners that make us think, 'why did you refer this patient with obvious dementia to a specialty memory clinic?' Then I try to make clear [to the resident] that there may be a legitimate reason, we are unaware of. Then I try to make clear that we should not immediately assume the general practitioner doesn't do their job or doesn't feel like doing it..' – Interview supervisor<br><br>**2. discuss roles and responsibilities of your own specialties within the patient's care network**<br>'the resident was struggling with: 'what did we contribute in the care for this patient?' Then we discussed how we made the uncertainty [that the general practitioner had] more explicit.[…] We have provided that general practitioner with support to go on for another year with this patient.' – interview Supervisor<br><br>'I find it helpful in supervision to be able to discuss and negotiate […]: what is and what isn't part of my tasks?' interview resident<br><br>**3. Formulate tasks for other specialties as part of collaborative care**<br>'Shall we ask the general practitioner to follow up on the hypertension?' – field note out patient consultation<br><br>'We are not going to follow up on that, [specialty] will have to take care of that themselves' – field note observation liaison consultation team
Barriers to IntraPC learning opportunities
**Perceived time constraints**
a. IntraPC is deprioritized, due to a perceived lack of time<br>b. A multitude of factors can lengthened RSDs, however RSDs with explored IntraPC learning opportunities were not substantially longer.
**Misalignment**
a. Feedback often remains implicit, its intention remains obscured<br>b. IntraPC learning outcomes are lost as residents interpret the feedback towards outcomes on medical knowledge acquisition or doctor-patient communication
**Monitoring-focus**
a. Residents checking diagnostic and treatment plans is most prevalent, learning is deprioritized<br>b. Supervisor mostly monitor patient safety and resident progress on medical knowledge<br>c. IntraPC learning is consistently deprioritized<br>d. When meaning making discussions on IntraPC leave treatment plans underdiscussed, a resident might feel frustrated and may not be open to IntraPC learning
**Entrustment struggle**
a. Residents tend to seek approval of the supervisor<br>b. Longer RSDs tend to be interpreted by residents as a sight limited entrustment and personal shortcoming, instead of meaning making discussions<br>c. Supervisors have to balance limiting supervision hours to indicate entrustment and creating reflective dialogues opportunities<br>d. IntraPC is regarded by supervisors as a more advanced learning objective, fit for residents with more medical technical entrusted activities<br>e. IntraPC is regarded by residents as a 'basic skill', more advanced resident no longer need to acquire.
