# Supplementary material for: An Offer You Can’t Refuse: Opportunities for Intraprofessional Collaboration Learning in the Workplace
Source: Perspect Med Educ. 2026 Jul 13;15(1):586–99. doi: 10.5334/pme.1863 (PMC13378418; doi:10.5334/pme.1863)
Supplement: Appendix 3. — Table 3. [file pme-15-1-1863-s3.pdf]

Appendix 3

Table 3: observation times comparing RSDs with and without utilized IntraPC learning moments per clinical setting

Work setting	Observations with utilized IntraPC learning opportunities<br><br>Observation ID – duration (min)	Observations without utilized IntraPC learning opportunities<br><br>Observation ID – duration (min)
In-patient acute care	3A – 26 min<br>14A – 24 min<br>14B – 32 min<br>20B – 115 min<br><br>Range: 24 – 115 min<br>Mean: 49 min	4A – 29 min<br>5A – 51 min<br>5B – 13 min<br>16A – 118 min<br>16B – 61 min<br>17A – 41 min<br>20A – 142 min<br><br>Range 13 – 142 min<br>Mean: 65 min
In-patient liaison team	2B – 11 min<br>6B – 26 min<br>11B – 35 min<br><br>Range 11 min – 35 min<br>Mean: 24 min	7A – 23 min<br>7C – 23 min<br>8A – 16 min<br>8B – 16 min<br>9A – 18 min<br>9B – 26 min<br>11A – 40 min<br>11B – 35 min<br>12A – 20 min<br>12B – 21 min<br><br>Range 16 min – 40 min<br>Mean: 24 min
Out-patient consultation	1B – 18 min<br>7D – 23 min<br>13B – 16 min<br>18A – 27 min<br>19A – 22 min<br>19B – 15 min<br><br>Range 16 min – 27 min<br>Mean 22 min	1A – 29 min<br>2A – 25 min<br>2C – 20 min<br>6A – 25 min<br>7B – 26 min<br>9C – 27 min<br>10A – 26 min<br>13A – 17 min<br>15A – 11 min<br>18B – 6 min<br>18C – 17 min<br>21A – 26 min<br>22A – 38 min<br>22B – 31 min<br><br>Range 6 min – 38 min<br>Mean 23 min
